# The acidic tumor microenvironment drives a stem-like phenotype in melanoma cells

**DOI:** 10.1007/s00109-020-01959-y

**Published:** 2020-08-15

**Authors:** Elena Andreucci, Silvia Peppicelli, Jessica Ruzzolini, Francesca Bianchini, Alessio Biagioni, Laura Papucci, Lucia Magnelli, Benedetta Mazzanti, Barbara Stecca, Lido Calorini

**Affiliations:** 1 Department of Experimental and Clinical Biomedical Sciences “Mario Serio”, Section of Experimental Pathology and Oncology, University of Florence, Florence, Italy; 2 Haematology Unit, Department of Experimental and Clinical Medicine, University of Florence, Florence, Italy; 3 Tumor Cell Biology Unit - Core Research Laboratory, Institute for Cancer Research, Prevention and Clinical Network (ISPRO), Florence, Italy; 4 Center of Excellence for Research, Transfer and High Education DenoTHE, University of Florence, Florence, Italy

**Keywords:** Acidosis, Tumor microenvironment, Melanoma, Cancer stem cells

## Abstract

**Abstract:**

Acidosis characterizes the microenvironment of most solid tumors and is considered a new hallmark of cancer. It is mainly caused by both “aerobic” and “anaerobic” glycolysis of differently adapted cancer cells, with the final product lactic acid being responsible of the extracellular acidification. Many evidences underline the role of extracellular acidosis in tumor progression. Among the different findings, we demonstrated that acidosis-exposed cancer cells are characterized by an epithelial-to-mesenchymal transition phenotype with high invasive ability, high resistance to apoptosis, anchorage-independent growth, and drug therapy. Acidic melanoma cells over-express SOX2, which is crucial for the maintenance of their oxidative metabolism, and carbonic anhydrase IX, that correlates with poor prognosis of cancer patients. Considering these evidences, we realized that the profile outlined for acid cancer cells inevitably remind us the stemness profile. Therefore, we wondered whether extracellular acidosis might induce in cancer cells the acquisition of stem-like properties and contribute to the expansion of the cancer stem cell sub-population. We found that a chronic adaptation to acidosis stimulates in cancer cells the expression of stem-related markers, also providing a high in vitro/in vivo clonogenic and trans-differentiating ability. Moreover, we observed that the acidosis-induced stem-like phenotype of melanoma cells was reversible and related to the EMT induction. These findings help to characterize a further aspect of stem cell niche, contributing to the sustainment and expansion of cancer stem cell subpopulation. Thus, the usage of agents controlling tumor extracellular acidosis might acquire great importance in the clinic for the treatment of aggressive solid tumor.

**Key messages:**

• Extracellular acidosis up-regulates EMT and stem-related markers in melanoma cells

• Acidic medium up-regulates *in vitro* self-renewal capacity of melanoma cells

• Chronic acidosis adaptation induces trans-differentiation ability in melanoma cells

• Melanoma cells adapted to acidosis show higher tumor-initiating potential than control cells

• Extracellular acidosis promotes a stem-like phenotype in prostate and colorectal carcinoma cells

## Introduction

Several evidences suggest that cancer stem cells (CSC) are the ultimate responsible of not only tumor initiation but also metastatic disease. CSC represent indeed the only cell population within the tumor bulk with tumor-initiating and self-renewal abilities, so that they are likely the unique cancer sub-population able to generate organ metastases [1].

Among several solid tumors, various reports indicate that melanomas are particularly enriched in CSC [2] and indeed considered one of the most aggressive cancers [3]. Melanoma cells display high phenotypic heterogeneity, expression of developmental genes, differentiation plasticity, and high tumorigenicity supporting the presence of a high number of CSC [4]. Melanoma cells often display some characteristics of neural cells [4] and are capable to organize vessel-like structures mimicking the endothelial cells, undergoing the so-called vasculogenic mimicry [5]. In specific conditions, melanoma cells may also acquire fibroblastic or adipogenic differentiation markers [6]. Quintana and colleagues reported that the incidence of tumorigenic cells in melanoma is greater over that observed for any other human cancer type [7]. Interestingly, by deeply investigating the tumorigenic and non-tumorigenic subpopulations, they did not find any significant phenotypic differences that could account for the diverse tumorigenic ability of these two populations [7]. They thus underlined the importance to investigate the role of epigenetic, genetic, and also environmental factors in inducing tumorigenicity. This represents an intriguing starting point to investigate how tumor microenvironment could influence cancer progression toward malignancy. An embryonic microenvironment has been shown to revert melanoma cells toward a more benign phenotype [8], while many evidences suggested that environmental factors such hypoxia and extracellular acidosis are critical for melanocyte transformation and progression toward malignancy [9–13].

Acidosis of tumor microenvironment is almost an obligatory step associated with the high proliferative rate of cancer cells exploiting glycolysis not only when oxygen tension reduces (anaerobic glycolysis) but even in the presence of sufficient oxygen to sustain oxidative phosphorylation (i.e., near blood vessels) (aerobic glycolysis or “Warburg effect”), with a subsequent lactic acid release [14]. Moreover, also the mitochondrial respiration, through the spontaneous or enzymatic CO_2_ hydration to carbonic acid, contributes to the extracellular medium acidification [15]. As a direct consequence, almost all solid tumors experience extracellular acidosis (ranging a pH from 6.4 to 7.0) [16], which is also caused by reduced lymphatic circulation and high interstitial pressure [17]. Many evidences highlighted the role of extracellular acidosis in the acquisition of tumor aggressive features, so that it is now recognized as a hallmark of cancer [9, 18, 19]. In the last years, we contributed to disclose most of the promoting effects of extracellular acidosis towards tumor malignancy, and the profile we delineated for the acid-adapted tumor cells inevitably reminds us the one proposed for CSC. Indeed, extracellular acidosis stimulates in various types of cancer several aspects related to stemness. Besides disclosing that acid-adapted melanoma cells overexpress well-known stem-related markers (i.e., SOX2—that sustains the oxidative metabolism exploited under acidosis [20]—and CAIX [21]—that, proposed as a marker of the pre-metastatic niche, always associates with resistant phenotype and correlates with very poor prognosis of tumor patients), we and other research groups demonstrated that extracellular acidosis induces the epithelial-to-mesenchymal transition (EMT) program [22–25], associated with a reduced proliferation rate, a high metalloprotease-dependent invasive ability [25, 26], and high apoptotic resistance in tumor cells [25, 27, 28]. Importantly, as we already reported [25], extracellular acidosis increases melanoma cell metastatic potential by triggering an “incomplete” or “partial” EMT program, that allows tumor cells to co-express mesenchymal and epithelial traits at the same time, and to be prompt to undergo the reverse mesenchymal-to-epithelial transition (MET) program at convenience during the metastatic cascade, a distinct sign of the high-plastic tumor phenotype induced by the acidic microenvironment. Another important trait of acidic cancer cell defining a high-resistant tumor phenotype is the anoikis resistance that we found significantly increased upon acidic exposure of melanoma cells [11]. Anoikis resistance is a crucial aspect for aggressive tumor cell subpopulation that, once detached from the primary mass, must survive into blood stream before their secondary lodgment. In addition, as a further indication of acidosis-induced resistant phenotype, acidic BRAF-mutated melanoma cells were found to not respond to targeted and standard chemotherapy, confirming the great impact of acidic tumor microenvironment in cancer progression [12].

Thus, we have investigated if an acidic tumor microenvironment could actually promote the expansion of the CSC subpopulation by inducing the acquisition of critical stem-like features in tumor cells. To investigate the influence of extracellular acidosis on stemness, cancer cells were chronically exposed to pH 6.7 ± 0.1 (which is the pH generally found in solid tumors, including melanoma) and considered “acid-adapted” when they recovered a proliferation rate similar to control cells maintained at standard pH [27, 29].

## Methods

### Cell cultures

Melanoma cell lines A375M6 [20] and M21 (kindly provided by Dr. Antony Montgomery, The Scripps Research Institute, La Jolla, CA), prostate cell line PC3 (purchased from the American Type Culture Collection, ATCC, Rockville, MD) and colon cancer cell line HCT116 (a kind gift of Dr. Matteo Lulli, Department of Clinical and Experimental Biomedical Sciences “Mario Serio”, University of Florence, Italy) [30], STR profiled and mycoplasma tested, were cultured in DMEM 4.5 g/l glucose, 2 mM L-glutamine, and 10% FBS (Euroclone, Milan Italy). The chronic extracellular acidic condition was mimicked in vitro as previously reported [11]. Briefly, chemical acidified medium was obtained by adding HCl 1 N in complete culture medium to reach pH 6.7 ± 0.1. pH value was monitored by using Orion pH meter 520A-1 at regular intervals during the first hour after the acidification to check the maintenance of correct pH of the medium. To obtain chronic acidic cultures, cancer cells were constantly subjected to a pH 6.7 medium for at least 3 months, until they recovered a similar growth rate as parent cells maintained at standard pH 7.4 condition. During the long-lasting acidic treatment, no significant death of cells was found.

### MTT assay

Cell viability was assessed using MTT (3-(4,5-dimethylthiazol-2-yl)-2,5-diphenyltetrazolium bromide) tetrazolium reduction assay (Sigma Aldrich, Milan, Italy) as previously described. Control or acid-adapted cancer cells were plated into 96-multiwell plates in complete medium without red phenol. After 24, 48, or 72 h the MTT reagent was added to the medium and plates were incubated at 37 °C. After 3 h, MTT was removed and the blue MTT–formazan product was solubilized with dimethyl sulfoxide (DMSO) (Sigma Aldrich). The absorbance of the formazan solution was read at 595 nm using the microplate reader (Bio-Rad, Milan, Italy).

### Flow cytometry

Cells were harvested by using Accutase (Euroclone, Milan, Italy), collected in flow cytometer tubes (2 × 10^5^ cells/tube), and stained 1 h at 4 °C with anti-CD133 (Affimetrix eBioscience, part of Thermo Fisher Scientific, Monza, Italy), anti-CD243 (Affimetrix eBioscience), anti-CD34 (BD PharMingen, San Diego, CA), anti-CD105 (Ancell, Bayport, MN), anti-CD73 (BD PharMingen), and anti-CD90 (BD PharMingen) antibodies. For intracellular antigen detection, cells were permeabilized for 15 min with 0.25% Tryton X-100 PBS, and then incubated 1 h at 4 °C with anti-ALDH1A1 (Abcam, Milan, Italy), anti-Nanog (GeneTex, CA, USA), anti-KLF4 (GeneTex), anti-OCT4 (GeneTex), and anti-SOX2 (GeneTex) antibodies. Cells were washed in PBS and incubated 1 h in the dark at 4 °C with secondary antibodies conjugated with FITC (Merk Millipore, Milan, Italy), Alexa Fluor 488 (Thermo Fisher Scientific, Monza Italy), PE (Immunotools, Germany), or APC (Immunotools). Samples were washed in PBS and analyzed at BD FACSCanto (BD Biosciences, Milan, Italy). The flow cytometer was calibrated using cells incubated with secondary antibody only. For each sample, 1 × 10^4^ events were analyzed.

### Tumorsphere formation assay

Control or acid-adapted cancer cells were plated at 150 cells/cm^2^ on a low-attachment 100 mm plate in DMEM/F12 supplemented with N2, 5 g/ml insulin, 20 ng/ml FGF-2, and 20 ng/ml EGF (all from Thermo Fisher Scientific) at standard pH. Cells were allowed to form tumorspheres by 10–15 days. For serial passaging, 10-day-old tumorspheres were dissociated in single cells with Accutase (Euroclone), and plated at 150 cells/cm^2^ on a low-attachment 100 mm plate for 10 additional days.

### In vitro limiting dilution assay

Control or acid-adapted cancer cells were seeded into ultra-low attachment 96-well plate at different cell doses, with a maximum of 100 cells per well and a minimum of one cell per well, and incubated in spheroid-forming conditions for 10 days at 37 °C, at standard pH. Colony formation was assessed by visual inspection. For each dilution series, we counted wells that showed sphere formation on day 11. Data were analyzed and displayed using the Extreme limiting dilution assay (ELDA) software available at http://bioinf.wehi.edu.au/software/elda/ [31].

### Western blotting analysis

Cells were washed with ice cold PBS containing 1 mM Na_4_VO_3,_ and lysed in 100 μl of cell RIPA lysis buffer (Merk Millipore) containing PMSF (Sigma-Aldrich), sodium orthovanadate (Sigma-Aldrich), and protease inhibitor cocktail (Merk Millipore) as previously described [11]. Aliquots of supernatants containing equal amounts of protein (50–100 μg) in Bolt LDS Sample Buffer (Thermo Fisher Scientific) were separated on Bolt® Bis-Tris Plus gels 4–12% precast polyacrylamide gels (Thermo Fisher Scientific). Fractionated proteins were transferred from the gel to a PVDF nitrocellulose membrane using an electroblotting apparatus (Bio-Rad, Segrate, MI, Italy). Blots were blocked for 1 h, at room temperature, with Odyssey blocking buffer (Dasit Science, Cornaredo, MI, Italy), and the membrane was probed at 4 °C overnight with primary antibodies diluited in a solution of 1:1 Odyssey blocking buffer/T-PBS buffer. The primary antibodies were mouse anti-Nanog, mouse anti-Oct4, mouse anti-NFkB p65 (1:500, GeneTex), mouse anti-Vimentin, rabbit anti-Zeb1 (1:1000, Santa Cruz Biotechnology, Santa Cruz, California), rabbit anti-Slug (1:500, Cell signaling Technology, Danvers, MA, US), rabbit anti-Snai1 (1:500, Biorbyt, Cambridge, United Kingdom), and mouse anti N-Cadherin (1:1000, DAKO Agilent, Milan, Italy). The membrane was washed in T-PBS buffer, incubated for 1 h at room temperature with goat anti-rabbit IgG Alexa Flour 750 antibody or with goat anti-mouse IgG Alexa Fluor 680 antibody (Invitrogen, Monza, Italy), and then visualized by an Odyssey Infrared Imaging System (LI-COR® Bioscience). Mouse anti-β-tubulin monoclonal antibody (Sigma, Saint Louis, MO, USA) was used to assess equal amount of protein loaded in each lane.

### siRNA transfection

For transfections, control siRNA (siCTRL, Thermo Fisher Scientific) and siRNA for RelA (NF-kB coding gene) (siRELA, Thermo Fisher Scientific) were diluted in Optimem medium (Thermo Fisher Scientific) to a final concentration of 5 nM and 50 nM for A375M6 and M21, respectively. Transfections were performed using Lipofectamine 3000 reagent (Thermo Fisher Scientific), following the manufacturer's instructions**.**

### Mouse xenograft assays

Six-week-old female CD-1 nude mice (Charles River, Calco, Lecco, Italy) were injected subcutaneously in lateral flanks with 10^2^ and 10^3^ A375M6 and M21 melanoma cells suspended in Matrigel (BD Biosciences)/DMEM (1:1) (DMEM at standard pH 7.4). Six animals for each cell line and dilution were used for the experiments, in order to get a statistically significant data and at the same time to accomplish to the 3R principle (Replacement, Reduction, and Refinement). Animals were monitored daily; subcutaneous tumor size was measured every 2–3 days by a caliper, and tumor volumes calculated using the formula *V* = 4/3π × *W*/2 × (*L*/2)^2^, where *W* and *L* are, respectively, tumor width (perpendicular tumor diameter) and length (largest tumor diameter). Mice were sacrificed before showing evident signals of discomfort with an overdose of isoflurane. Statistical analysis of tumor take was performed using the ELDA software [31]. Experiments with animals were conducted in accordance with national guidelines and were approved by the ethics committee of the Animal Welfare Office of the Italian Ministry of Health (n°401/2015/PR) and conformed to the legal mandates and Italian guidelines for the care and maintenance of laboratory animals.

### Adipocyte and osteocyte melanoma cell differentiation

Sub-confluent melanoma cells seeded in 6-well plate were treated with pro-adipogenic differentiating medium—DMEM 1 g/L glucose supplemented with 10% FBS (Euroclone), 0.5 mM isobutyl methylxanthine, 1 μM dexamethasone, 10 μg/ml of insulin, and 70 μM indomethacin (Sigma Aldrich)—or pro-osteogenic differentiating medium—DMEM 1 g/L glucose supplemented with 10% FBS (Euroclone), 10 nM dexamethasone, 100 μg/ml ascorbic acid, and 10 mM β-glycerophosphate (Sigma Aldrich)—for 3 weeks. Pro-adipogenic and pro-osteogenic differentiating media were prepared and administered at standard pH. Media were replaced every other day. Lipid drops of adipogenic-differentiated melanoma cells were stained by Oil Red O (Sigma Aldrich): briefly, cells were fixed for 30 min at room temperature in 4% formaldehyde, then the stock solution (30 mg Oil Red O powder/10 ml isopropanol) was diluted 3:2 (V:V) in deionized H_2_O and fixed cells stained for 5 min. Calcium deposits of osteoblast-like-differentiated melanoma cells were stained with Alizarin Red (Sigma Aldrich): briefly, cells were fixed in 70% ethanol for 1 h at 4 °C and stained for 10 min with 40 mM Alizarin Red solution in deionized H_2_O at pH 4.2.

To quantify adipocyte and osteoblast-like differentiation of melanoma cells, qPCR analysis was performed for pro-adipogenic (LPL, CEBPα and PPARγ) and pro-osteogenic (ALPL, COL1A1, DMP1, and SOST) differentiation genes.

### Quantitative real-time PCR

Total RNA was prepared using Tri Reagent (Sigma-Aldrich), agarose gel checked for integrity, and reverse transcribed with iScript cDNA Synthesis Kit (Bio-Rad) according to the manufacturer’s instructions. Selected genes were evaluated by a real-time qPCR with 7500 Fast Real-Time PCR System (Applied Biosystems, Monza, Italy). Fold change was determined by the comparative Ct method calculating the average of β-actin, β2-microglobulin, TATA-Box Binding Protein (TBP), and 18s used as reference genes. Amplification was performed with the PCR setting: 40 cycles of 95 °C for 15 s and of 60 °C for 60 s using PowerUp SYBR Green Master Mix (Thermo Fisher Scientific). Primer sequences (IDT, Tema Ricerca, Bologna, Italy) are listed in Table 1 (β-actin, β2-microglobulin, TBP, and 18s used as reference genes).

**Table 1:** Primer sequences for real-time PCR analysis

Gene	Forward (5′-3′)	Reverse (5′-3′)
β2-microglobulin	GCCGTGTGAACCATGTGACT	GCTTACATGTCTCGATCCCACTT
β-actin	TCGAGCCATAAAAGGCAACT	CTTCCTCAATCTCGCTCTCG
18s	CGCCGCTAGAGGTGAAATTCT	CGAACCTCCGACTTTCGTTCT
ΤΒP	CAACAGCCTGCCACCTTAC	CTGAATAGGCTGTGGGGTC
N-cadherin	CACTGCTCAGGACCCAGAT	TAAGCCGAGTGATGGTCC
E-cadherin	CGGGAATGCAGTTGAGGATC	AGGATGGTGTAAGCGATGGC
Twist	CGGGAGTCCGCAGTCTTA	TGAATCTTGCTCAGCTTGTC
Snail	CCCAGTGCCTCGACCACTAT	CCAGATGAGCATTGGCAG
Vimentin	TGTCCAAATCGATGTGGATGTTTC	TTGTACCATTCTTCTGCCTCCTG
LPL	TCCGCGTGATTGCAGAGA	GCTCGTGGGAGCACTTCACT
CEBPα	GGGTCTGAGACTCCCTTTCCTT	CTCATTGGTCCCCCAGGAT
PPARγ	TCAGGGCTGCCAGTTTCG	GCTTTTGGCATACTCTGTGATCTC
ALPL	CCGTGGCAACTCTATCTTTGG	GATGGCAGTGAAGGGCTTCTT
COL1A1	CTGTTCTGTTCCTTGTGTAACTGTGTT	GCCCCGGTGACACATCAA
DMP1	ACATTGAGATAGAGAGCCGGAAA	TGGTCCCCAATGGGTTTGT
SOST	TCAGAGGAGGCAGAAATGGAA	CCACACCGCTCCCTTAAAAC

### Statistical analysis

The experiments were performed at least five times for a reliable application of statistics. All samples used were included in the statistical analysis. Statistical analysis was performed by *T* test, One-way analysis of variance (ANOVA), and Two-way ANOVA with GraphPad Prism 6 software, as specified in each figure legend. Statistical significances were accepted at *p* < 0.05. Values are presented as mean of independent experiments ± SD.

## Results

### Extracellular acidosis up-regulates EMT and stem-related markers in melanoma cells

**Fig. 1:**
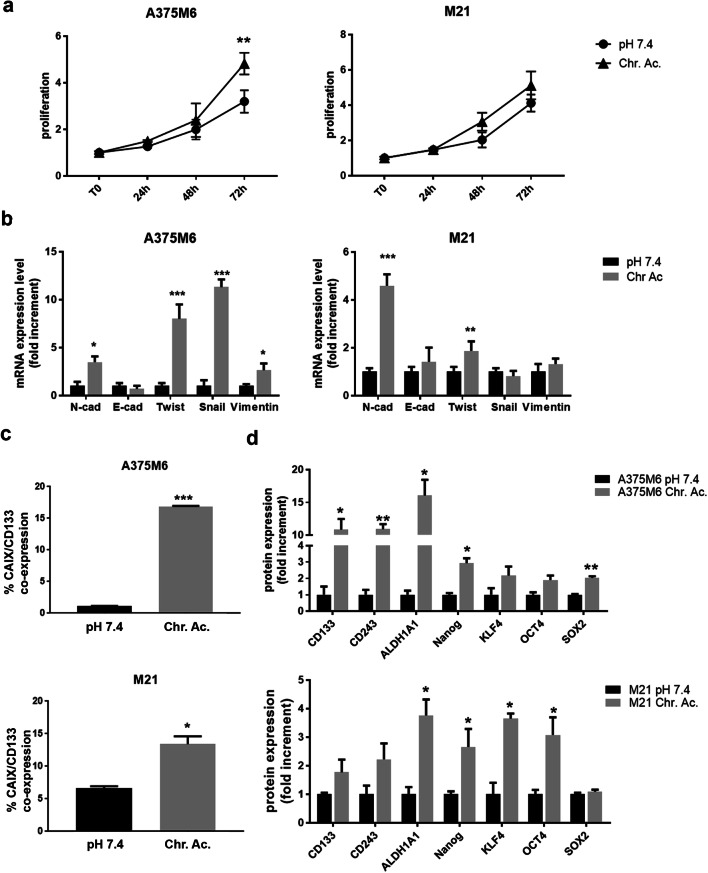
EMT- and stem-related markers of melanoma cells chronically exposed to extracellular acidosis. **a** Growth curves of acid-adapted (Chr.ac.) and control (pH 7.4) A375M6 and M21 cells obtained by MTT assay. **b** Real-time PCR of EMT-related markers in acid-adapted and control A375M6 and M21 cells. **c** Flow cytometer analysis of CAIX and CD133 co-expressing cells under standard and acidic conditions. **d** Flow cytometer analysis of stem-related markers in A375M6 and M21 cells exposed to extracellular acidosis compared with control. *p < 0.05, **p < 0.01, ***p < 0.001, Two-way ANOVA Sidak's multiple comparisons test for **a**, **b**, and **d**; *t* test for **c**

A375M6 and M21 melanoma cells were exposed to pH 6.7 for approximately 3 months and considered “acid-adapted” when they recovered a proliferation rate similar to control cells maintained at standard pH (Fig. 1a). Chronic adaptation to extracellular acidosis, as well as acute exposure [25], induces a partial EMT program, a feature related to stemness [32], by maintaining the expression levels of the epithelial marker E-cadherin and at the same time inducing the acquisition of the mesenchymal markers N-cadherin and Twist in both A375M6 and M21 cell lines, Snail and Vimentin in only A375M6 cells (Fig. 1b).

Acid-adapted cells, previously shown to over-express CAIX [21]—a marker of CSC strongly associated with drug resistance [33, 34]— also show a higher percentage of CD133-CAIX co-expressing cells than control (Fig. 1c). Following these findings, we evaluated, by flow cytometry analysis, the expression of a panel of stem-related proteins, observing a significant increase of CD133, CD243, ALDH1A1, NANOG, and SOX2 for acid-adapted A375M6 and of ALDH1A1, NANOG, KLF4, and OCT4 for acid-adapted M21 cells compared with control (Fig. 1d).

### Extracellular acidosis up-regulates self-renewal capacity of melanoma cells in vitro

**Fig. 2:**
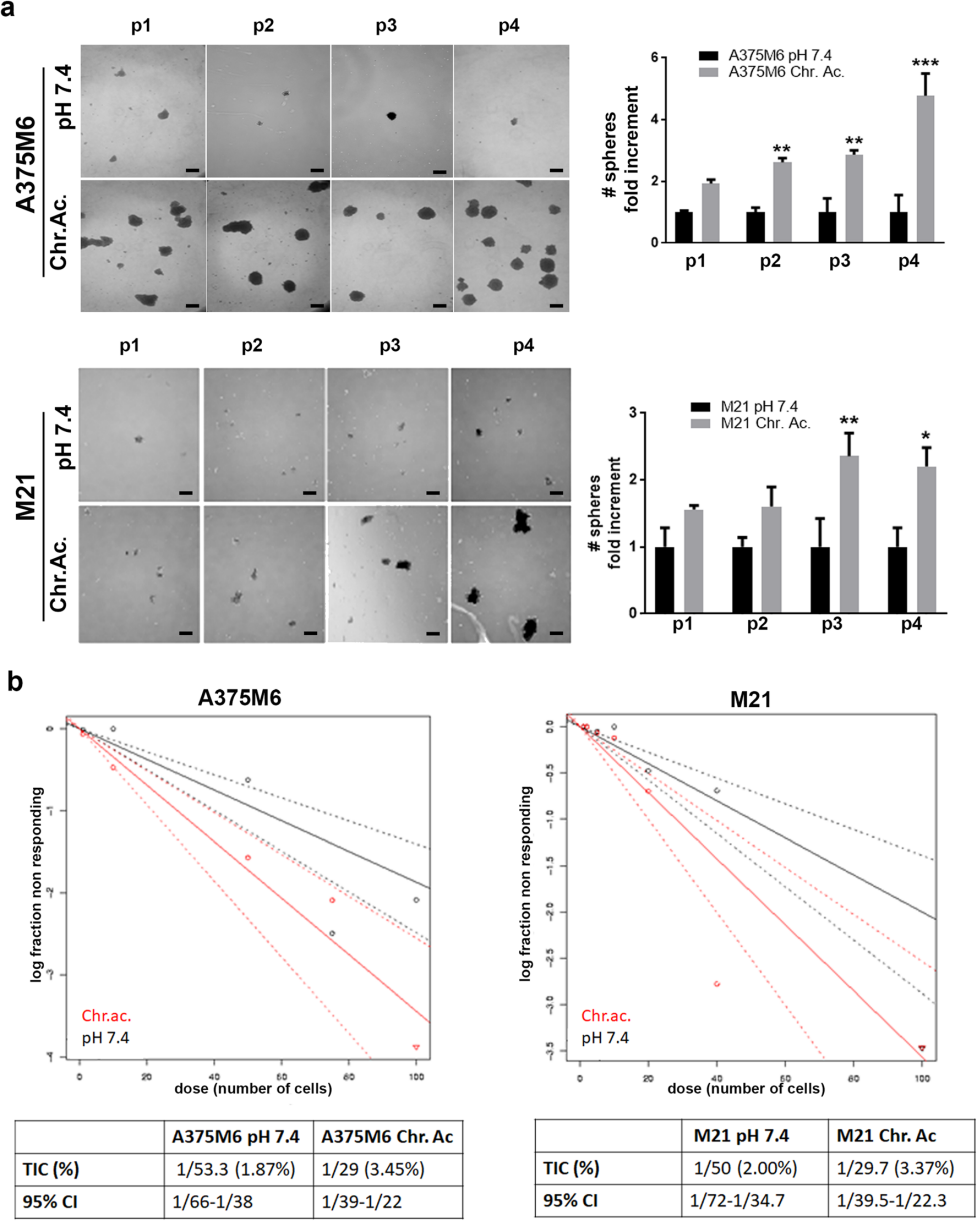
In vitro self-renewal ability of melanoma cells chronically exposed to extracellular acidosis. **a** Tumorsphere formation assay of A375M6 and M21 cells under chronic extracellular acidosis (Chr.ac.) and standard condition (pH 7.4). Representative images (left) and relative quantification chart (right) of spheres from passage 1 to 4 (p1–p4). Scale bar: 200 μm. **b** Limiting dilution sphere assay of acid-adapted and control A375M6 and M21 cells. ELDA analysis plot (upper) and relative table reporting the TIC percentage (lower). *p < 0.05, **p < 0.01, ***p < 0.001, Two-way ANOVA, Sidak's multiple comparisons test

One of the most important property of CSC is the self-renewal potential, tested in vitro as the ability of melanoma cells to form tumorspheres. Acid-adapted A375M6 and M21 cells gave rise to higher number of melanospheres than control, and such ability is further enhanced along with the serial passages (Fig. 2a). These results were confirmed using a limiting dilution assay, where acid-adapted or control cells were evaluated for sphere formation 10 days later cell plating. By extreme limiting dilution analysis (ELDA) software analysis [31], acid-adapted melanoma cells showed a significant increase in tumor-initiating cells (TIC), from 18.7 to 33.7% for A375M6 and from 20 to 33.6% for M21 cells, suggesting that the low pH is able to expand the self-renewal population (Fig. 2b).

### Reversibility of the acidosis-induced stem-like phenotype in melanoma cells

**Fig. 3:**
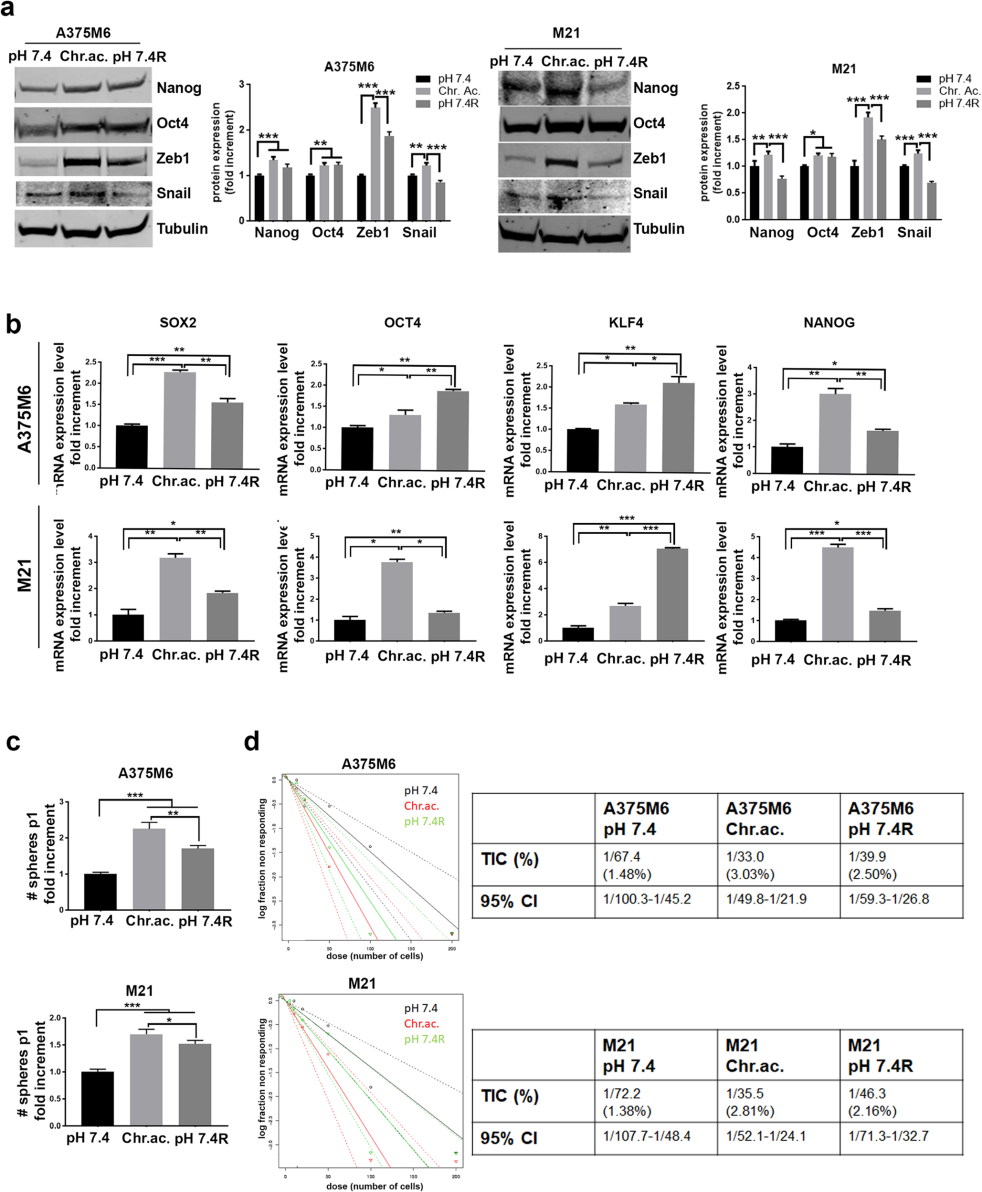
The acidosis-induced stem-like phenotype is partially reversible by restoring standard pH condition. **a** WB analysis of EMT- and stem-related markers in control (pH 7.4), acid-adapted (Chr.ac.), and pH 7.4 restored (pH 7.4R) A375M6 and M21 cells. **b** Real-time PCR of stem-related markers in control, acid-adapted, and pH 7.4 restored A375M6 and M21 cells. Tumorsphere formation (**c**) and limiting dilution sphere (**d**) assays of control, acid-adapted and pH 7.4 restored A375M6 and M21 cells. *p < 0.05, **p < 0.01, ***p < 0.001; two-way ANOVA, Sidak's multiple comparisons test for **a**; one-way ANOVA, Tukey's multiple comparisons test for **b** and **c**

Since CSC have been described as dynamic, able to exhibit plasticity and to revert their stem cell properties [35], we grown back acid-adapted melanoma cells in standard pH medium for 15 days and obtained tumor subpopulations thereafter referred to as “pH 7.4 restored” (pH 7.4R). By western blot analysis, we observed that the expression levels of the stem-related markers Nanog and Oct4, significantly increased in acid-adapted melanoma cells, did not vary in pH 7.4R A375M6, while Nanog expression was significantly reduced in pH 7.4R M21 (Fig. 3a). Interestingly, in both pH 7.4R A375M6 and M21 cells, we observed a clear reduction of ZEB1 and Snail (Fig. 3a), two EMT-activators that are also known as stemness mediators [36, 37]. By real-time PCR analysis, we observed a tendence of stem-related genes, that were found up-regulated in acid-adapted melanoma cells, to come back near to the control level, in particular SOX2 and Nanog in both A375M6 and M21 cells, OCT4 in only M21 cell line; on the contrary, KFL4 was not only maintained at high level as acid-adapted cells but was even increased in both pH 7.4R cell lines (Fig. 3b). Moreover, the pH 7.4R melanoma cells showed a significantly reduced tumorsphere formation ability (Fig. 3c) and a decreased TIC percentage (Fig. 3d) compared with acid-adapted melanoma cells, despite still increased compared with control cells. These data suggest that the stem-like phenotype of melanoma cells induced by acidosis is reversible, but 15 days is not enough to reach a complete phenotype reversion.

### EMT is required for the acquisition of the acidosis-induced stem-like phenotype in melanoma cells

**Fig. 4:**
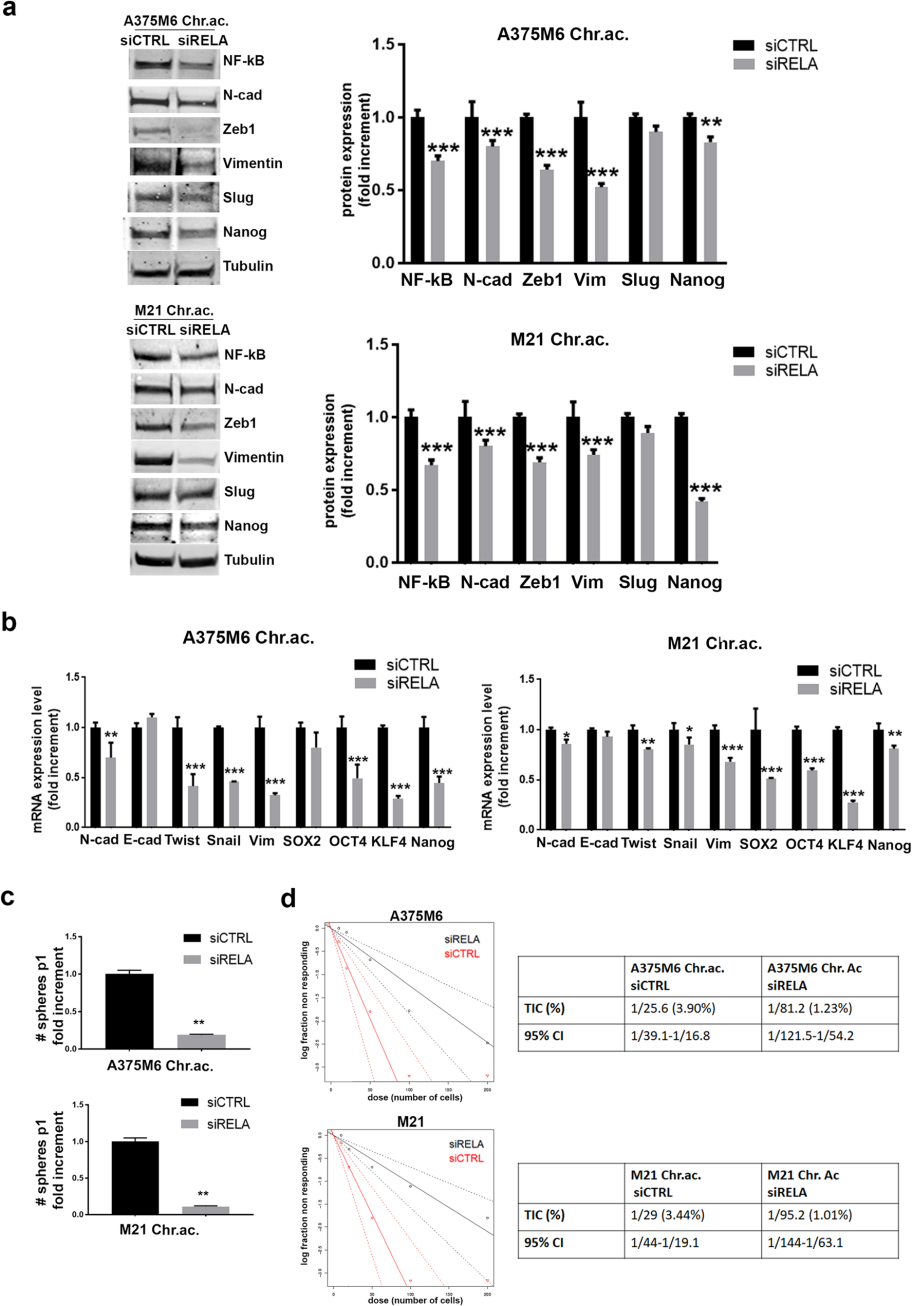
EMT down-regulation via RELA silencing in acid-adapted melanoma cells reduces the stem-like phenotype. WB (**a**) and real-time PCR (**b**) analysis of EMT- and stem-related markers in siCTRL and siRELA acid-adapted A375M6 and M21 cells. Tumorsphere formation (**c**) and limiting dilution sphere (**d**) assays of siCTRL and siRELA acid-adapted A375M6 and M21 cells. *p < 0.05, **p < 0.01, ***p < 0.001; two-way ANOVA, Sidak's multiple comparisons test for **a** and **b**; *t* test for **c**

Since recent papers revealed the fundamental role of EMT in stemness induction [32], we wondered if the induction of an EMT phenotype in acid-adapted melanoma cells was required for the acquisition of stemness features. The NF-kB transcriptional factor is known to regulate EMT in different type of cancers [38, 39], and, in addition, we previously reported that it drives the acidosis-induced EMT in melanoma cells [25]. We thus decided to down-regulate acidosis-induced EMT by silencing the NF-kB coding gene RelA and observed, by western blot analysis, a clear reduction of the EMT markers N-Cadherin, Zeb1, Vimentin, and Slug (even though its reduction was not significant), and of the stem-related marker Nanog (Fig. 4a). The real-time PCR analysis confirmed the reduction in mRNA expression of the EMT markers N-Cadherin, Twist, Snail, and Vimentin and of the stem-related markers SOX2, OCT4, KFL4, and Nanog (Fig. 4b). Finally, the tumor sphere formation (Fig. 4c) and the in vitro limiting dilution (Fig. 4d) assays, respectively, showed a reduced melanosphere formation and a decreased TIC percentage in both the cell lines tested. These data suggest that the EMT program is required for the acquisition of a stem-like phenotype by acid-adapted melanoma cells.

### Melanoma cells adapted to extracellular acidosis show higher tumor-initiating potential than control cells

**Fig. 5:**
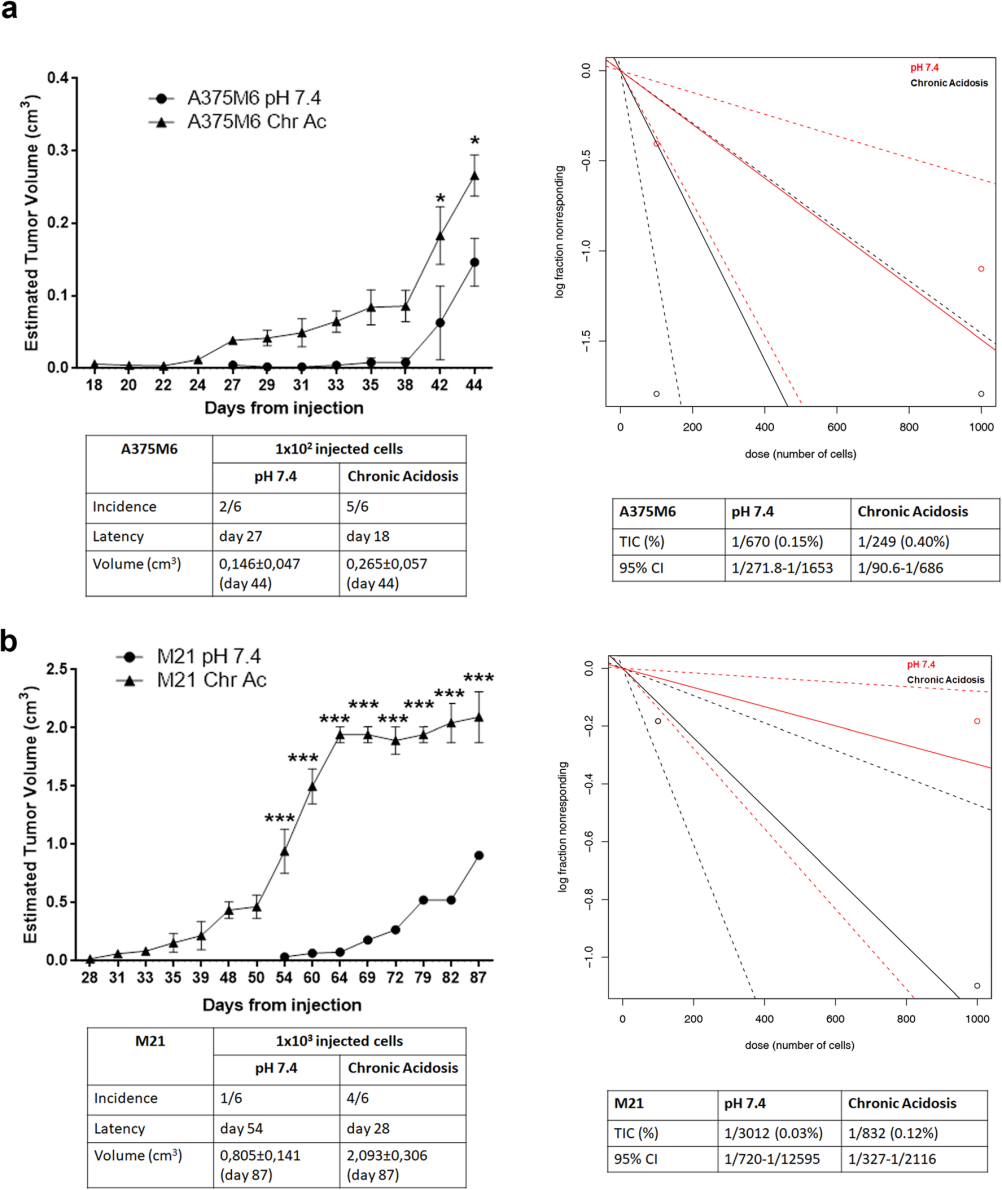
Extracellular acidosis increases in vivo tumor initiation and growth of melanoma cells. In vivo tumor growth (left) and estimated tumor-initiation frequency (ELDA software) (right) after subcutaneous injection in CD-1 mice of either 100 acid-adapted (Chr.ac.) or standard (pH 7.4) A375M6 cells (**a**) or 1000 acid-adapted or standard M21 cells (**b**). **p < 0.01, ***p < 0.001; two-way ANOVA, Sidak's multiple comparisons test (pH 7.4 vs Chr.ac. for each time point)

Tumor-initiation ability in vivo was tested by subcutaneously injecting in CD-1 nude mice a low number of standard and acid-adapted A375M6 and M21 melanoma cells and allowed to generate tumor masses. By injecting 10^2^ acid-adapted A375M6 (Fig. 5a), nodules developed in higher number and earlier compared with control (5/6 tumors at day 18 vs 2/6 tumors at day 27, respectively), and also increased in tumor volume (Fig. 3a, left panel). The ELDA analysis indicated that acid-adapted group contains higher percentage of TIC than control (0.40% vs 0.15%, respectively) (Fig. 5a, right panel). By injecting 10^3^ A375M6 cells, no significant differences were observed between control and acid-adapted group (4/6 tumors at day 20 vs 5/6 tumors at day 22, respectively; data not shown), suggesting that acid-adapted A375M6 melanoma cells contain an expanded TIC subpopulation emerging when using a low number of cancer cells.

Thereafter, injecting 10^2^ control or acid-adapted M21 melanoma cells, a similar and very low incidence was obtained (1/6 tumors of both groups), although latency (day 50 for acid-adapted vs day 60 for control group) and tumor volume evaluation indicated a more effectiveness of acid-adapted cells than control (data not shown). Increasing the number of injected M21 cells up to 10^3^, significant differences between the two groups have been observed in terms of incidence (4/6 tumors for acid-adapted vs 1/6 tumors for control group), latency (day 28 for acid-adapted vs day 54 for control group), and tumor volume (Fig. 5b, left panel). The ELDA analysis (Fig. 5b, right panel) clearly showed that the percentage of TIC of acid-adapted group is higher than in control one (0.12% vs 0.03%, respectively).

### Chronic acidosis adaptation induces high plasticity and trans-differentiation ability in melanoma cells

**Fig. 6:**
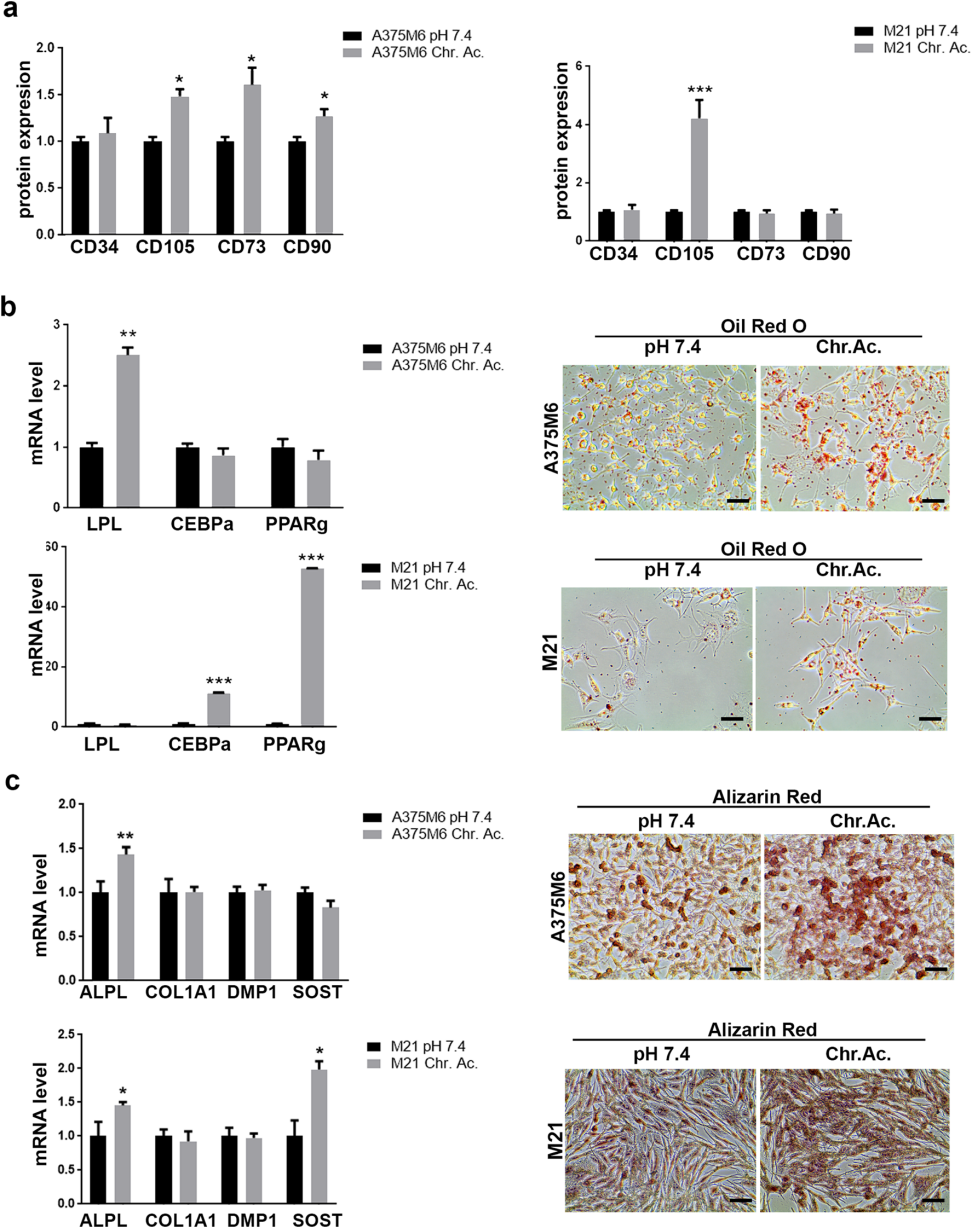
Chronic acidosis adaptation induces high plasticity and trans-differentiation ability in melanoma cells. **a** Flow cytometer analysis for the expression of the mesenchymal markers CD105, CD73, and CD90 in acid-adapted (Chr.ac.) A375M6 and M21 compared with relative control. CD34 used as negative control. **b** Real-time PCR analysis for adipogenic differentiation markers LPL, CEBPα, and PPARγ (left) and representative pictures of Oil Red O lipid drop staining (right) in acid-adapted A375M6 and M21 compared with control cells. **c** Real-time PCR analysis for osteogenic differentiation markers ALPL, COL1A1, DMP1, and SOST (left) and representative pictures of Alizarin Red calcium phosphate deposit staining (right) in acid-adapted A375M6 and M21 compared with control cells. Scale bar: 50 μm. *p < 0.05, **p < 0.01, ***p < 0.001; two-way ANOVA, Sidak's multiple comparisons test

A different way to test in vitro stemness of tumor cells is to evaluate their ability to trans-differentiate in other cellular types when subjected to specific differentiating stimuli. To this aim, standard and acid-adapted melanoma cells were cultured for 3 weeks under pro-adipogenic- or pro-osteogenic-differentiating media and then evaluated by molecular and histological techniques. We firstly checked by flow cytometry their intrinsic predisposition to express the CD105, CD73, and CD90 mesenchymal stem cell markers, and the CD34 hematopoietic stem cell marker was used as negative control, observing that chronic acidosis increased the expression of CD105, CD73, and CD90 in A375M6 and CD105 in M21 cells compared with control cells (Fig. 6a). Treating melanoma cells for 3 weeks with pro-adipogenic-differentiating medium, we observed a higher trans-differentiation ability of acid-adapted cells compared with control cells, assessed via qPCR analysis of the adipogenic markers LPL, CEBPα, and PPARγ, and via Oil Red O staining of intracytoplasmic lipid drops (Fig. 6b). In parallel, we observed that the pro-osteogenic-differentiating medium induces a stronger positivity for the Alizarin Red staining in acid-adapted melanoma cells compared with control cells, despite at a molecular level only ALPL (for A375M6 and M21 cells) and SOST (for M21 cells) genes were significantly increased in acidic-adapted cells (Fig. 6c). These findings disclose the ability of acid-adapted A375M6 and M21 melanoma cells to easily trans-differentiate towards mesoderm-derived lineages upon specific stimuli.

### Extracellular acidosis promotes a stem-like phenotype in prostate and colorectal carcinoma cells

**Fig. 7:**
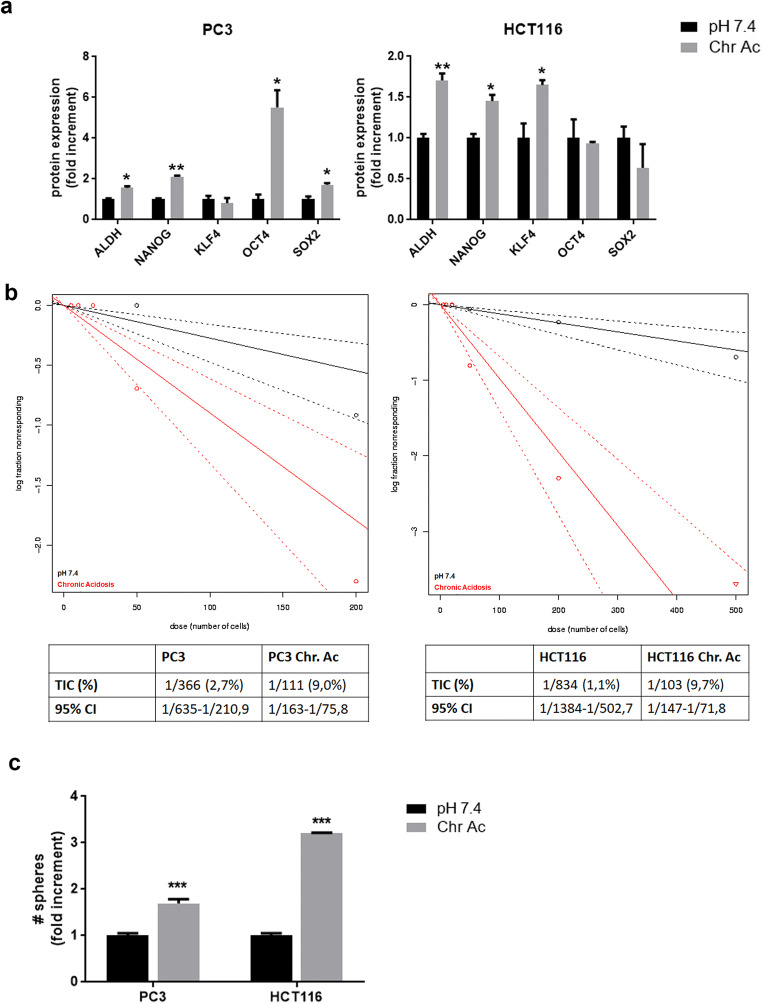
Stem-like features of prostate and colorectal cancer cells chronically exposed to extracellular acidosis. **a** Flow cytometer analysis for stem-related markers in PC3 prostate and HCT116 colorectal cancer cells exposed to chronic acidosis (Chr.ac.) compared with control (pH 7.4). **b** Limiting dilution sphere assay of acid-adapted and control PC3 and HCT116 cells. ELDA analysis plot (upper panel) and relative TIC percentage value (lower panel). **c** Tumorsphere formation assay of PC3 and HCT116 cells under chronic extracellular acidosis and standard condition *p < 0.05, **p < 0.01, ***p < 0.001; two-way ANOVA, Sidak's multiple comparisons test.

To verify whether a chronic low pH might be also able to extend the stem-cell subpopulation not only in melanoma but also in other tumor types, we evaluated some aspects of CSC in prostate and colorectal adenocarcinoma cell lines, PC3 and HCT116, respectively, chronically exposed to an acidic medium compared with control. First of all, we observed that acid-adapted prostate and colon cancer cells, compared with control, express higher protein levels of stem-related markers (Fig. 7a). Moreover, limiting dilution analysis shows that acid-adapted PC3 and HCT116 cells display an increased self-renewal potential, suggested by the higher sphere frequency and the TIC percentage obtained by ELDA analysis (Fig. 7b). In line with these results, acid-adapted PC3 and HCT116 cells also gave rise to higher number of tumorspheres compared with control (Fig. 7c), highlighting the possibility that acidosis promotion of stemness is a more general phenomenon that could be extended to different tumor types.

## Discussion

Many types of cancer display subpopulations of CSC, that share features with physiological stem cells (i.e., self-renewal, differentiation, indefinite proliferation abilities) through which they cause tumor progression [40–42]. CSC are protected and maintained in a well-defined microenvironment providing a permissive niche able to enforce their survival ability and aggressive properties [43]. Alongside hypoxia, that plays an important role in stemness [44], extracellular acidosis might represent another crucial characteristic of tumor microenvironment able to influence stemness of cancer cells.

We collected a wide number of evidences inevitably linking a possible association between chronic extracellular acidosis and the promotion of a stem-like phenotype in melanoma cells.

Notably, EMT, apoptosis resistance, anoikis, and drug resistance features, we already observed under acidic conditions, have been linked to a tumor profile with successful metastatic dissemination [6, 45, 46]. Here we demonstrate that a higher number of acid-adapted melanoma cells co-express CAIX and CD133 proteins compared with control, a further indication of acidosis-induced stemness. Indeed, CAIX expression has been correlated with stem cell niches in normal tissues [47] and considered a critical mediator of the self-renewal and invasive potential of cancer cells by regulating the transcription of EMT- and stemness-related genes [33, 48].

Here we show the induction of the self-renewal and pluripotency markers Nanog, KLF4, OCT4, and SOX2 and the over-expression of the CSC markers CD133, CD243, and ALDH1A1 in melanoma cells exposed to chronic acidosis compared with control cells. In line with our results, acidosis has been associated with the increased CSC-marker expression in prostate cancer [49] and malignant glioma [50, 51], osteosarcoma [52], and the maintenance of stemness in mesenchymal cells [53, 54]. Also, OCT-4 expression in murine fibroblasts increases as pH shifts from 7.4 to 6.5 [55].

We confirmed that extracellular acidosis induces the self-renewal capability of cancer cells by the in vitro tumorsphere formation and limiting dilution assays. We found that acid-adapted cells give a higher number of melanospheres with bigger size than control cells and preserve their stem-like phenotype for at least four passages, whereas control cells gradually lose their ability to form melanospheres. This effect is not limited to melanoma, but rather proved in PC3 prostate and HCT116 colon cancer cells, that indeed also showed high expression of stem-related proteins.

We also showed that the stem-like phenotype induced in melanoma cells by acidosis is a reversible phenomenon, as suggested by the reduced expression of stem-related markers and by the lower self-renewal capability of acid-adapted cells grown back for 15 days at standard pH medium, compared with acid-adapted cells. The phenotype we observed was not completely reverted at control level, but the phenomenon was clear enough to confirm that the acidosis-induced stem-like phenotype is dynamic and subjected to variations depending on environmental conditions, an evidence that contributes to define the increased plasticity and aggressiveness of acid-adapted tumor cells [35].

Several studies have demonstrated the link between EMT and stemness in a variety of human carcinoma [32, 45], and recently, EMT with its mediators, such as Zeb1 [36], Snail [37], Slug [56], and NF-kB [57, 58], has been described as a key mechanism by which cells are conferred with stem-cell properties. Here, we demonstrated that the stem-like phenotype induced in acid-adapted melanoma cells is EMT-dependent, since by inhibiting the well-known EMT mediator NF-kB, the stem-like phenotype acquired by acid-adapted melanoma cells has clearly been reduced.

CSC are also denominated tumor-initiating cells (TIC) being responsible for the initiation, recurrence, and metastases of a tumor. For that, increased in vivo tumorigenicity is one of the hallmarks of CSC. Interestingly, melanoma TIC population has been demonstrated to depend on SOX2 expression [59], that we found up-regulated under acidic conditions [20]. The in vivo evaluation of the tumor-initiation ability of acid-adapted melanoma cells compared with control showed that chronic acidosis induces the earlier development of a higher number of tumors compared with the standard pH condition, and the ELDA statistical analysis revealed a higher percentage of TIC in acid-adapted group than control. Some evidences showed that increasing the extracellular pH reduces tumor growth, prevents cancer invasion [26, 60], and restores drug sensitivity [61]. Recently acidosis has been proposed as potential therapeutic target to eradicate CSC [62].

Another hallmark of CSC is the multi-lineage differentiation plasticity that influences tumor progression and drug resistance [63]. Based on the origin of melanoma, we thought that the melanoma CSC should potentially differentiate into mesenchymal cell types, such as adipocytes and osteoblast-like cells. By treating melanoma cells with appropriate differentiating media, we observed that acid-adapted melanoma cells, compared with control, show higher ability to trans-differentiate in adipocyte and osteoblast-like cells. A recent study showed that a continuous exposure to low pHe for 21 days of bone marrow stem cells and dental pulp stem cells impairs the osteogenic differentiation of both cell types [53], suggesting that extracellular acidosis is crucial for the maintenance of a de-differentiated state.

To conclude, our results provide evidences that acidity leads to a reprogramming of a very plastic tumor phenotype toward the expression of stem-related markers, high clonogenic, and trans-differentiating ability. This could clarify the reasons why extracellular acidosis is able to promote aggressive traits in cancer, dramatically contributing to tumor progression and metastatic disease. Thus, acidosis needs to be considered a new and specific cue of stem cell niche, able to offer an innovative target for cancer therapy.

## Abbreviations

CAIX: Carbonic anhydrase IX
CSC: Cancer stem cells
ELDA: Extreme limiting dilution analysis
EMT: Epithelial-to-mesenchymal transition
TIC: Tumor-initiating cells

## References

[1] Peitzsch C, Tyutyunnykova A, Pantel K, Dubrovska A (2017). Cancer stem cells: the root of tumor recurrence and metastases. Semin Cancer Biol.

[2] Seftor EA, Margaryan NV, Seftor REB, Hendrix MJC (2019). Heterogeneity of Melanoma with Stem Cell Properties. Adv Exp Med Biol.

[3] Siegel R, Ma J, Zou Z, Jemal A (2014). Cancer statistics, 2014. CA Cancer J Clin..

[4] Fang D, Nguyen TK, Leishear K, Finko R, Kulp AN, Hotz S, van Belle PA, Xu X, Elder DE, Herlyn M (2005). A tumorigenic subpopulation with stem cell properties in melanomas. Cancer Res.

[5] Anurag C, Singh N, Vipin Kumar G, Verma M (2019). Vasculogenic Mimicry and Its Role in Cancer. Am J Pharmacol.

[6] Schatton T, Frank MH (2008). Cancer stem cells and human malignant melanoma. Pigment Cell Melanoma Res.

[7] Quintana E, Shackleton M, Sabel MS, Fullen DR, Johnson TM, Morrison SJ (2008). Efficient tumour formation by single human melanoma cells. Nature..

[8] Postovit L-M, Seftor EA, Seftor REB, Hendrix MJC (2006). Influence of the microenvironment on melanoma cell fate determination and phenotype. Cancer Res.

[9] Webb BA, Chimenti M, Jacobson MP, Barber DL (2011). Dysregulated pH: a perfect storm for cancer progression. Nat Rev Cancer.

[10] Peppicelli S, Andreucci E, Ruzzolini J, Laurenzana A, Margheri F, Fibbi G, del Rosso M, Bianchini F, Calorini L (2017). The acidic microenvironment as a possible niche of dormant tumor cells. Cell Mol Life Sci..

[11] Peppicelli S, Ruzzolini J, Bianchini F, Andreucci E, Nediani C, Laurenzana A (2019). Anoikis resistance as a further trait of acidic-adapted melanoma cells. J Oncol..

[12] Ruzzolini J, Peppicelli S, Andreucci E, Bianchini F, Margheri F, Laurenzana A, Fibbi G, Pimpinelli N, Calorini L (2017). Everolimus selectively targets vemurafenib resistant BRAFV600E melanoma cells adapted to low pH. Cancer Lett.

[13] Muz B, de la Puente P, Azab F, Azab AK (2015). The role of hypoxia in cancer progression, angiogenesis, metastasis, and resistance to therapy. Hypoxia (Auckl).

[14] Vander Heiden MG, Cantley LC, Thompson CB (2009). Understanding the Warburg effect: the metabolic requirements of cell proliferation. Science..

[15] Mookerjee SA, Goncalves RLS, Gerencser AA, Nicholls DG, Brand MD (2015). The contributions of respiration and glycolysis to extracellular acid production. Biochim Biophys Acta.

[16] Reshetnyak YK, Yao L, Zheng S, Kuznetsov S, Engelman DM, Andreev OA (2011). Measuring tumor aggressiveness and targeting metastatic lesions with fluorescent pHLIP. Mol Imaging Biol.

[17] Vaupel P (2004). Tumor microenvironmental physiology and its implications for radiation oncology. Semin Radiat Oncol.

[18] Damaghi M, Gillies R (2017). Phenotypic changes of acid-adapted cancer cells push them toward aggressiveness in their evolution in the tumor microenvironment. Cell Cycle.

[19] Pillai SR, Damaghi M, Marunaka Y, Spugnini EP, Fais S, Gillies RJ (2019). Causes, consequences, and therapy of tumors acidosis. Cancer Metastasis Rev.

[20] Andreucci E, Pietrobono S, Peppicelli S, Ruzzolini J, Bianchini F, Biagioni A, Stecca B, Calorini L (2018). SOX2 as a novel contributor of oxidative metabolism in melanoma cells. Cell Commun Signal.

[21] Andreucci E, Peppicelli S, Carta F, Brisotto G, Biscontin E, Ruzzolini J, Bianchini F, Biagioni A, Supuran CT, Calorini L (2017). Carbonic anhydrase IX inhibition affects viability of cancer cells adapted to extracellular acidosis. J Mol Med.

[22] Riemann A, Rauschner M, Gießelmann M, Reime S, Haupt V, Thews O (2019). Extracellular acidosis modulates the expression of epithelial-mesenchymal transition (EMT) markers and adhesion of epithelial and tumor cells. Neoplasia.

[23] Suzuki A, Maeda T, Baba Y, Shimamura K, Kato Y (2014). Acidic extracellular pH promotes epithelial mesenchymal transition in Lewis lung carcinoma model. Cancer Cell Int.

[24] Zhu S, Zhou HY, Deng SC, Deng SJ, He C, Li X, Chen JY, Jin Y, Hu ZL, Wang F, Wang CY, Zhao G (2017). ASIC1 and ASIC3 contribute to acidity-induced EMT of pancreatic cancer through activating Ca2+/RhoA pathway. Cell Death Dis.

[25] Peppicelli S, Bianchini F, Torre E, Calorini L (2014). Contribution of acidic melanoma cells undergoing epithelial-to-mesenchymal transition to aggressiveness of non-acidic melanoma cells. Clin Exp Metastasis.

[26] Estrella V, Chen T, Lloyd M, Wojtkowiak J, Cornnell HH, Ibrahim-Hashim A, Bailey K, Balagurunathan Y, Rothberg JM, Sloane BF, Johnson J, Gatenby RA, Gillies RJ (2013). Acidity generated by the tumor microenvironment drives local invasion. Cancer Res.

[27] Persi E, Duran-Frigola M, Damaghi M, Roush WR, Aloy P, Cleveland JL, Gillies RJ, Ruppin E (2018). Systems analysis of intracellular pH vulnerabilities for cancer therapy. Nat Commun.

[28] Ryder C, McColl K, Zhong F, Distelhorst CW (2012). Acidosis promotes Bcl-2 family-mediated evasion of apoptosis: involvement of acid-sensing G protein-coupled receptor Gpr65 signaling to Mek/Erk. J Biol Chem.

[29] Wojtkowiak JW, Rothberg JM, Kumar V, Schramm KJ, Haller E, Proemsey JB, Lloyd MC, Sloane BF, Gillies RJ (2012). Chronic autophagy is a cellular adaptation to tumor acidic pH microenvironments. Cancer Res.

[30] Andreucci E, Ruzzolini J, Peppicelli S, Bianchini F, Laurenzana A, Carta F, Supuran CT, Calorini L (2019). The carbonic anhydrase IX inhibitor SLC-0111 sensitises cancer cells to conventional chemotherapy. J Enzyme Inhib Med Chem.

[31] Hu Y, Smyth GK (2009). ELDA: extreme limiting dilution analysis for comparing depleted and enriched populations in stem cell and other assays. Journal of Immunological Methods.

[32] Fabregat I, Malfettone A, Soukupova J (2016). New insights into the crossroads between EMT and stemness in the context of cancer. J Clin Med..

[33] Ledaki I, McIntyre A, Wigfield S, Buffa F, McGowan S, Baban D, Li JL, Harris AL (2015). Carbonic anhydrase IX induction defines a heterogeneous cancer cell response to hypoxia and mediates stem cell-like properties and sensitivity to HDAC inhibition. Oncotarget.

[34] Supuran CT, Alterio V, Di Fiore A, D’Ambrosio K, Carta F, Monti SM (2018). Inhibition of carbonic anhydrase IX targets primary tumors, metastases, and cancer stem cells: three for the price of one. Med Res Rev.

[35] Sugihara E, Saya H (2013). Complexity of cancer stem cells. Int J Cancer.

[36] Preca BT, Bajdak K, Mock K, Sundararajan V, Pfannstiel J, Maurer J, Wellner U, Hopt UT, Brummer T, Brabletz S, Brabletz T, Stemmler MP (2015). A self-enforcing CD44s/ZEB1 feedback loop maintains EMT and stemness properties in cancer cells. Int J Cancer.

[37] Hojo N, Huisken AL, Wang H, Chirshev E, Kim NS, Nguyen SM, Campos H, Glackin CA, Ioffe YJ, Unternaehrer JJ (2018). Snail knockdown reverses stemness and inhibits tumour growth in ovarian cancer. Sci Rep.

[38] Huber MA, Azoitei N, Baumann B, Grünert S, Sommer A, Pehamberger H, Kraut N, Beug H, Wirth T (2004). NF-kappaB is essential for epithelial-mesenchymal transition and metastasis in a model of breast cancer progression. J Clin Invest.

[39] Nomura A, Majumder K, Giri B, Dauer P, Dudeja V, Roy S, Banerjee S, Saluja AK (2016). Inhibition of NF-kappa B pathway leads to deregulation of epithelial-mesenchymal transition and neural invasion in pancreatic cancer. Lab Invest.

[40] Dean M, Fojo T, Bates S (2005). Tumour stem cells and drug resistance. Nat Rev Cancer.

[41] Korkaya H, Wicha MS (2010). Cancer stem cells: nature versus nurture. Nat Cell Biol.

[42] Reya T, Morrison SJ, Clarke MF, Weissman IL (2001). Stem cells, cancer, and cancer stem cells. Nature.

[43] Plaks V, Kong N, Werb Z (2015). The cancer stem cell niche: how essential is the niche in regulating stemness of tumor cells?. Cell Stem Cell.

[44] Yun Z, Lin Q (2014). Hypoxia and regulation of cancer cell stemness. Adv Exp Med Biol.

[45] Shibue T, Weinberg RA (2017). EMT, CSCs, and drug resistance: the mechanistic link and clinical implications. Nat Rev Clin Oncol.

[46] Mani SA, Guo W, Liao M-J, Eaton EN, Ayyanan A, Zhou AY (2008). The epithelial-mesenchymal transition generates cells with properties of stem cells. Cell.

[47] Liao S-Y, Lerman MI, Stanbridge EJ (2009). Expression of transmembrane carbonic anhydrases, CAIX and CAXII, in human development. BMC Dev Biol.

[48] Lock FE, McDonald PC, Lou Y, Serrano I, Chafe SC, Ostlund C (2013). Targeting carbonic anhydrase IX depletes breast cancer stem cells within the hypoxic niche. Oncogene.

[49] Huang S, Tang Y, Peng X, Cai X, Wa Q, Ren D, Li Q, Luo J, Li L, Zou X, Huang S (2016). Acidic extracellular pH promotes prostate cancer bone metastasis by enhancing PC-3 stem cell characteristics, cell invasiveness and VEGF-induced vasculogenesis of BM-EPCs. Oncol Rep.

[50] Filatova A, Seidel S, Böğürcü N, Gräf S, Garvalov BK, Acker T (2016). Acidosis acts through HSP90 in a PHD/VHL-independent manner to promote HIF function and stem cell maintenance in glioma. Cancer Res.

[51] Hu P, Li S, Tian N, Wu F, Hu Y, Li D, Qi Y, Wei Z, Wei Q, Li Y, Yin B, Jiang T, Yuan J, Qiang B, Han W, Peng X (2019). Acidosis enhances the self-renewal and mitochondrial respiration of stem cell-like glioma cells through CYP24A1-mediated reduction of vitamin D. Cell Death Dis.

[52] Avnet S, Di Pompo G, Chano T, Errani C, Ibrahim-Hashim A, Gillies RJ (2017). Cancer-associated mesenchymal stroma fosters the stemness of osteosarcoma cells in response to intratumoral acidosis via NF-κB activation. Int J Cancer..

[53] Massa A, Perut F, Chano T, Woloszyk A, Mitsiadis TA, Avnet S (2017). The effect of extracellular acidosis on the behaviour of mesenchymal stem cells in vitro. Eur Cell Mater.

[54] Hazehara-Kunitomo Y, Hara ES, Ono M, Aung KT, Komi K, Pham HT, Akiyama K, Okada M, Oohashi T, Matsumoto T, Kuboki T (2019) Acidic pre-conditioning enhances the stem cell phenotype of human bone marrow stem/progenitor cells. Int J Mol Sci 20. 10.3390/ijms20051097 PMC6429188 30836626

[55] Som A, Bloch S, Ippolito JE, Achilefu S (2016). Acidic extracellular pH of tumors induces octamer-binding transcription factor 4 expression in murine fibroblasts in vitro and in vivo. Sci Rep.

[56] Guo W, Keckesova Z, Donaher JL, Shibue T, Tischler V, Reinhardt F, Itzkovitz S, Noske A, Zürrer-Härdi U, Bell G, Tam WL, Mani SA, van Oudenaarden A, Weinberg RA (2012). Slug and Sox9 cooperatively determine the mammary stem cell state. Cell.

[57] Zakaria N, Mohd Yusoff N, Zakaria Z, Widera D, Yahaya BH (2018). Inhibition of NF-κB signaling reduces the stemness characteristics of lung cancer stem cells. Front Oncol.

[58] Xiang T, Long H, He L, Han X, Lin K, Liang Z, Zhuo W, Xie R, Zhu B (2015). Interleukin-17 produced by tumor microenvironment promotes self-renewal of CD133+ cancer stem-like cells in ovarian cancer. Oncogene.

[59] Santini R, Vinci MC, Pandolfi S, Penachioni JY, Montagnani V, Olivito B, Gattai R, Pimpinelli N, Gerlini G, Borgognoni L, Stecca B (2012). Hedgehog-GLI signaling drives self-renewal and tumorigenicity of human melanoma-initiating cells. Stem Cells.

[60] Silva AS, Yunes JA, Gillies RJ, Gatenby RA (2009). The potential role of systemic buffers in reducing intratumoral extracellular pH and acid-mediated invasion. Cancer Res.

[61] Wojtkowiak JW, Verduzco D, Schramm KJ, Gillies RJ (2011). Drug resistance and cellular adaptation to tumor acidic pH microenvironment. Mol Pharm.

[62] Vander Linden C, Corbet C (2019). Therapeutic targeting of cancer stem cells: integrating and exploiting the acidic niche. Front Oncol.

[63] Kemper K, de Goeje PL, Peeper DS, van Amerongen R (2014). Phenotype switching: tumor cell plasticity as a resistance mechanism and target for therapy. Cancer Res.

